# Freshwater influx to the Eastern Mediterranean Sea from the melting of the Fennoscandian ice sheet during the last deglaciation

**DOI:** 10.1038/s41598-022-12055-1

**Published:** 2022-05-19

**Authors:** Tristan Vadsaria, Sébastien Zaragosi, Gilles Ramstein, Jean-Claude Dutay, Laurent Li, Giuseppe Siani, Marie Revel, Takashi Obase, Ayako Abe-Ouchi

**Affiliations:** 1 Laboratoire des Sciences du Climat et de l’Environnement, CEA-CNRS-Université Paris Saclay, Gif-sur-Yvette, France; 2 Atmosphere and Ocean Research Institute, The University of Tokyo, 5-1-5, Kashiwanoha, Kashiwa, Chiba 277-8568 Japan; 3 UMR-CNRS 5805 EPOC, Université de Bordeaux, Allée Geoffroy Saint-Hilaire, Pessac, France; 4 Laboratoire de Météorologie Dynamique, CNRS-ENS-Ecole Polytechnique-Sorbonne Université, Paris, France; 5 GEOPS, UMR 8148 Université Paris-Saclay, Orsay, France; 6 Université de la Côte d’Azur, CNRS, OCA, IRD, Geoazur, Valbonne, France

**Keywords:** Ocean sciences, Palaeoceanography, Palaeoclimate, Climate sciences

## Abstract

Between the Last Glacial Maximum and the mid-Holocene, the Mediterranean Sea experienced major hydrological changes. The deposition of the last sapropel, S1, during the Early Holocene is a consequence of these changes. In order to cause anoxia in the Eastern Mediterranean Sea (EMS) bottom water, a long preconditioning period of a few thousand years would need to occur throughout the deglaciation prior to S1. It is generally believed that this freshwater was of North Atlantic origin, later supplemented by the African Humid period (AHP). Here, we investigate another potentially important source of freshwater to the EMS: the Fennoscandian ice sheet (FIS) meltwater, running into the Caspian and Black Seas. A few scenarios of continental hydrologic perturbation have been developed to drive a high-resolution Mediterranean Sea general circulation model. We demonstrate that, during the last deglaciation, FIS meltwater flowing into the Black Sea reduced surface salinity and ventilation over the main convection areas in the EMS. By including continental hydrological changes, a more consistent framework is produced to characterize the hydrology of the Mediterranean Sea during the last deglaciation and the Early Holocene.

## Introduction

Sapropel occurrence in the Mediterranean has a strong correlation with regional hydrologic perturbations driven by precession cycles of the Earth’s orbit and is characterized by increased African monsoon run-off and enhanced freshwater input from the Nile River^1,2^. Recent sapropels, such as S1 and S5, have been shown to be initiated by the precession cycle (~ 21 ka periodicity) and modulated by the glacial-interglacial cycle (100 ka)^3^. A modeling investigation successfully simulated the protracted oxygen depletion leading to the S1 onset (~ 3.5 ka in the Ionian and the Levantine basin, for instance) within a numerical Mediterranean general circulation model coupled with its biogeochemical components^4^. A preconditioning period throughout the deglaciation is believed essential for bottom water anoxia to occur. The massive influx of low-salinity Atlantic water from Heinrich stadial 1 (HS1, 18–15.5 ka), entering the Mediterranean Sea through the Gibraltar Strait, combined with high biological productivity, drastically modified the ventilation and caused the development of anoxia within the Eastern Mediterranean Sea (EMS)^4^.

The Gibraltar Strait, however, may not be the only route by which the deglaciation affected the EMS. A complementary source could be continental. Fennoscandian ice sheet (FIS) meltwater could have reduced the salinity of the Black Sea^5–8^ and the Marmara Sea^9,10^, ultimately impacting the Aegean Sea’s surface salinity, stratification and convection.

Here, based on recent findings in Eastern European deglaciation hydrology^5,7,10–16^, we elaborate different scenarios of continental hydrologic perturbations affecting the EMS. Using a numerical model of the Mediterranean Sea, we further quantify their impact and discuss their plausibility in light of proxy-evidence.

**Figure 1:**
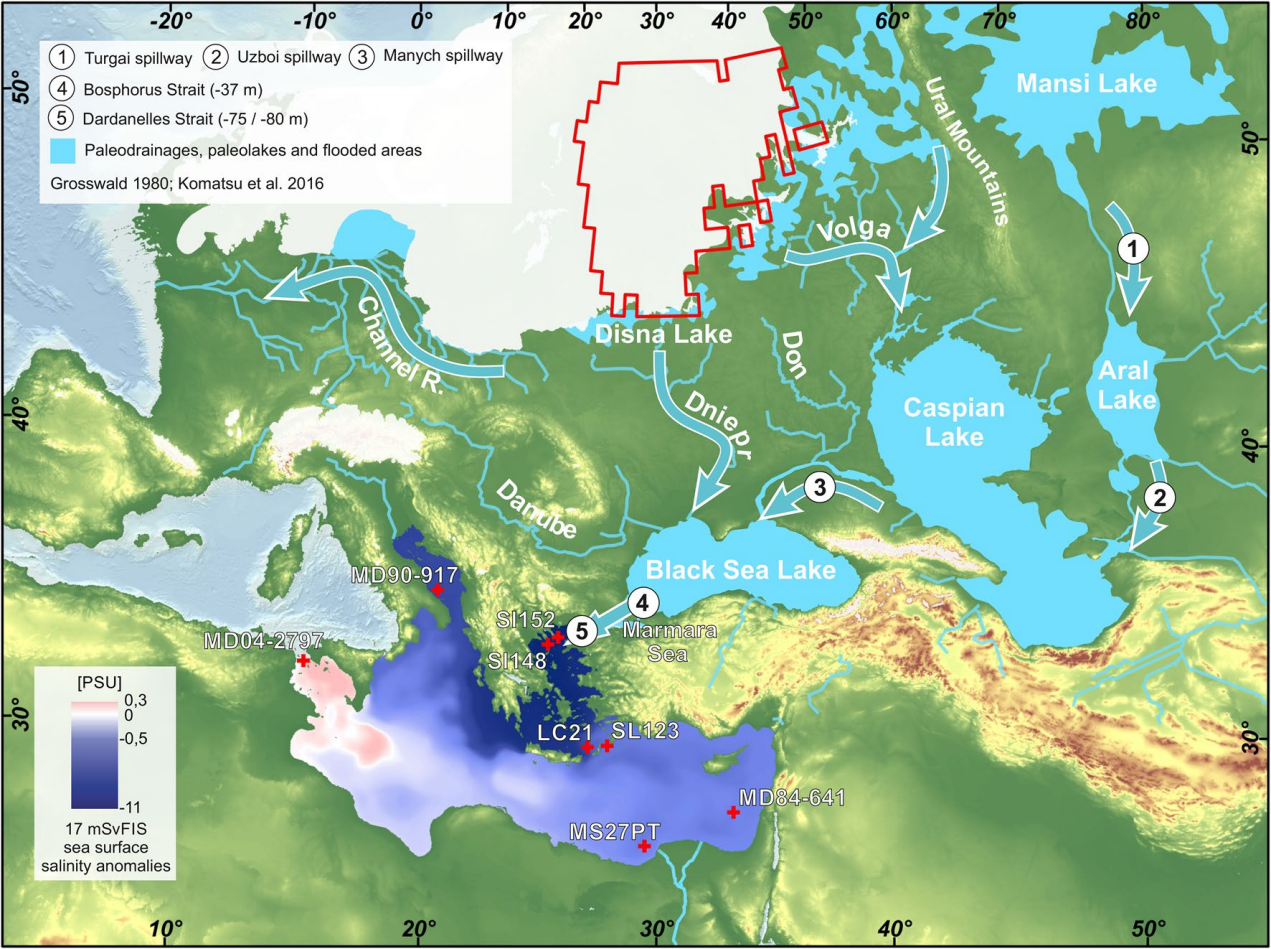
Map of LGM ice caps including the Fennoscandia Ice Sheet^67^ and its southward drainage basin with major rivers indicated, including the Volga, Don, and Dnieper^68,69^. The red contour shows the surface area (1,119,166 km^2^) from which meltwater potentially flows into the Black Sea and Caspian Sea. The anomalous sea surface salinity from the “17mSvFIS” experiment (averaged over the last 10 years) is overlaid for the Mediterranean. Crosses indicate the location of marine cores evoked/used in this study: SL152 (40°05.19′N, 24° 36.65′E; water depth: 978 m)^15,21^, SL148 (39°45.23′N, 24°05.78′E; water depth: 1094 m)^18–20^, LC21 (35°39.7′N, 26°35.0′E; water depth: 1520 m)^3^, SL123 (35°45.3′N, 27°33.3′E; water depth: 728 m)^20^, MD84-641 (33°02′N, 33°38′E; water depth: 1375 m)^58^, MS27PT (31°47′N, 29°27′E; water depth: 1389 m)^40,41^, MD90-917 (41°17′N, 17°36′E; water depth: 1010 m)^55^ and MD07-2797 (36°57′N, 11°40′E; water depth: 771 m)^56,57^. The map has been generated using ArcGIS. Bathymetric data sets come from the GEBCO 2014 bathymetric grid (both ocean and land terrain, https://www.gebco.net).

Sediments and stratigraphy evidence from the Caspian basin show that large flooding fed by FIS melting occurred (in Ponto-Caspian) from 17 to 10 ka, with an overflow to the Black Sea from 16 to 14 ka^13^ (Fig. 1). This finding was further confirmed by studies suggesting that the Ponto-Caspian basin collected meltwater and trapped fine-grained sediment transported from the southeastern margin of the FIS during the deglaciation (mainly through the Dnieper and Volga rivers)^7^.

Isotopic studies on the Black Sea (also referred to as the ‘Neoeuxine Lake’) reveal a northern freshwater source but diverge on the timing, attributing it to either a series of meltwater pulses^17^ between 16.5 and 14.8 ka BP or repeated seasonal bursts of ~ 220 years from cyclic drainage of Lake Disna over successive periods between 17.5 and 15.5 ka^6^. Sediment studies further suggest massive meltwater input to the Black Sea between 16.4 and 15.4 ka BP (greater than 25,000 km^3^)^8^. It is suggested that this meltwater volume would fill both the Caspian an Black Seas^8^, leading to flooding and outflowing. Regarding stratification, a different timing is proposed: the Black Sea could have overflowed during HS1, associated with low stratification of the basin^5^, or during the Younger Dryas (YD, 12.8–11.6 ka)^17^. The study of Major et al.^14^ combining proxy data and shallow- or deep-sill (Bosphorus) scenarios proposes the following chronology. Between 25 and 13.4 ka, the Black Sea overflowed for the two sill types. From 13.4 to 12.8 ka, it overflowed for the deep-sill model but not for the shallow-sill model. Between 12.8 and 11 ka, the Black Sea did not overflow for the shallow model while it is “possible” that it did for the deep-sill model. Between 11 and 10 ka, overflowing was likely for the shallow-sill model and reasonably certain for the deep-sill model^14^. A recent reconstruction of Black Sea paleosalinity suggests an overflow to the Marmara Sea from 23 to 14.7 ka, but not during the Bølling‐Allerød (BA, 14.7–12.9 ka) and the YD, due to surface salinity increase (associated with higher evaporation) of the Black Sea^16^.

In the Marmara Sea, data for the last deglaciation are scant. However, it appears, based on δ^18^O records, that a hydrologic connection persisted from the LGM to the end of the YD, likely indicating an outflow to the Marmara Sea, assuming a Black Sea level (BSL) close to the Bosphorus sill depth^9^. It is also proposed that freshwater inflow to the Marmara Sea could occur from 50 ka and persist continuously until 14.7 ka^10^.

Conversely, oceanic proxy data studies over the Aegean Sea do not seem to converge regarding substantial freshwater input from the Black Sea during the last deglaciation. The LC21 core, located in the south of the Aegean Sea with a continuous δ^18^O record from 150 ka BP to present, shows a drop of ~ − 2‰ in δ^18^O during HS1 (between 16 and 15 ka). However, the authors of the study do not firmly attribute this decrease to meltwater inputs^3^. It is also interesting to mention a rich compilation of cores in the Aegean Sea that suggest a reduction in local deep-water formation and the prevalence of eutrophic conditions at the SL148 core (in the north) during the BA. This condition was attributed to increased riverine nutrient flux^18^, probably due to incoming deglaciation meltwater from the northern borderland mountains (Rhodope glacier)^19,20^ or increased precipitation in the northern borderland for the SL152 core (North Aegean)^21^. In the SL123 core (South Aegean), a relatively small reduction in deep water oxygenation is recorded during the BA^20^. However, a recent reconstruction of sea surface salinity (SSS) in the SL152 core showed a decrease of ~ − 1.5 PSU between 11 and 10 ka associated with the freshwater outburst from Lake Agassiz^15^.

Finally, the recent study by Aksu and Hiscott^11^ reviewed the available data over the last 20 years and proposed a multi proxy analysis of the BSL and Marmara Sea level during the last deglaciation. They find a Black Sea outflow to the Aegean Sea during 17.2–15.7 cal ka over the Bosphorus and the Dardanelles sills due to the FIS melting. During the BA, based on the Dardanelles sill depth and Marmara sea-level reconstructions, the first incursion of Aegean/Mediterranean water into the Marmara Sea would be dated at 14.7 ka^9^ (with a sill depth of – 80 m^22^) or at 13.8 ka (− 75 m)^11^. Over this period and until the end of the YD at least, the Aegean Sea flowed into the Marmara Sea. After 12.5 ka, outburst floods from lakes in the Altay and Sayan Mountains of Central Asia may have contributed to the BSL rise, leading to outflowing into the Aegean Sea from 11.1 to 10.2 ka^11^.

In summary, several studies based on paleo data indicate that the Caspian and Black Seas were filled with FIS meltwater either through outburst/flooding (likely from 17 to 10 ka for the Caspian Sea and between 17.5 and 14.8 ka for the Black Sea) or through continuous melting until the YD. Moreover, the Black Sea collected the majority of freshwater from the Caspian Sea^7,13,23,24^.

A review of previous studies on the Marmara and Black Seas in the period 21–10 ka suggests that FIS meltwater would lead to an outflow from the Black Sea to the Aegean Sea, at least from the LGM to 14.7 ~ 13.4 ka firstly, and then from 12 to 11 to 10 ka. During most of the BA and YD, an outflow to the Aegean Sea is unlikely due to higher evaporation leading to increased salinity of the Black Sea (potentially associated with a lower BSL).

Aegean Sea studies suggest an inflow during the BA, but linked to nearby regional meltwater inputs. This mismatch between studies focusing on the north of the Dardanelles Strait on the one hand, and the south on the other hand, shows that there is still a lack of understanding of the chronology and quantification of potential freshwater outflow from the Black Sea during the last deglaciation.

Our goal is to revisit the hypothesis of freshwater brought by glacial melting of FIS to the EMS (Fig. 1). Using high-resolution regional oceanic modeling, we assess the impact of this meltwater discharge into the EMS and discuss to what extent proxy data can attest to this Black Sea freshwater outflowing.

## Results

### Freshwater flux reconstruction from Fennoscandia to the Bosphorus Strait

To investigate the effect of FIS melting on the EMS via Black Sea freshening, we use a three-step approach. First, we quantify the meltwater flux from the FIS into the Caspian and Black Seas catchments using ice sheet reconstructions^25–27^. Second, by using transient simulations of the last deglaciation, we estimate the water budget over the Black Sea for this period. Deducting this budget from the FIS meltwater, we compute the BSL evolution and the outflow over the Bosphorus sill. Third, we use numerical modeling to examine the effect of the meltwater on the SSS, stratification and ventilation of the EMS. The high spatial resolution of the Mediterranean Oceanic Regional Circulation Model (ORCM), NEMOMED8, does not allow us to perform transient simulations from the LGM to Early Holocene. To overcome this issue, we extract the most significant phases of the Black Sea outflowing based on proxy studies. Then, we imposed the fluxes as boundary conditions on the ORCM^28^ and ran different experiments (of 100 years each) (see the “Materials and methods” section for a detailed description of ice sheet reconstruction and experimental design). Finally, we put the Black Sea outflow into perspective regarding ORCM output and the proxy data which show freshwater perturbation affecting the EMS.

The three ice sheet reconstructions we used, ICE-6G^26^, GLAC-1D^27^ and Patton et al.^25^, rely on different methods. ICE-6G uses Global Positioning System measurements and space geodesic constraints. GLAC-1D reconstruction is based on ice-sheet modeling using climate simulations. Patton et al.’s reconstruction uses a thermomechanical model with regional constraints.

**Figure 2:**
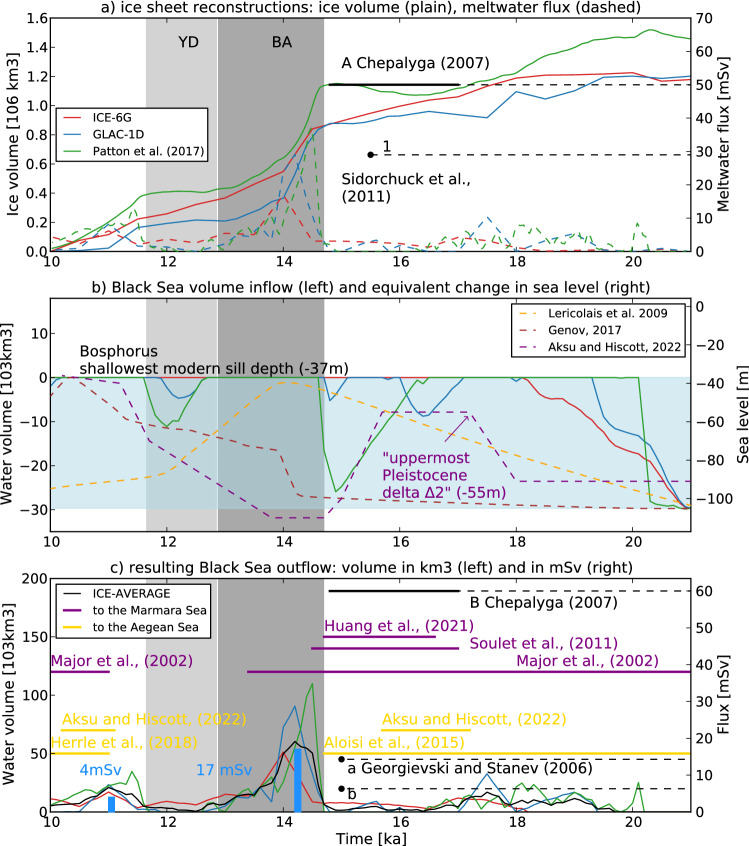
(**a**) Ice sheet reconstructions from three different sources with the volume of ice (plain curves, left axis) and associated meltwater flux (dashed curves, right axis) potentially draining to the Caspian Sea and Black Sea catchment. “A”^13^ and “1”^12^ represent meltwater-driven flux reconstructions from the Caspian Sea to the Black Sea. (**b**) Black Sea level (right axis, m, relative to modern sea level) and associated volume changes (left axis, km^3^) deduced from the Black Sea’s water budget with FIS meltwater input as shown in panel **a**), and the regional water flux P–E (precipitation-minus-evaporation) from two transient simulations of MIROC4m and Trace-21 ka. The water volume is expressed as the complementary volume needed to reach the Bosphorus sill at − 37 m, the level necessary for the Black Sea to outflow to the Mediterranean. The lowest BSL at the LGM is estimated to be – 105 m^29,30^ and the total volume for outflowing to occur is 29,675 km^3^ (light blue horizontal patch). Superimposed are BSL reconstructions by Lericolais et al.^29^, Genov^30^ and Aksu and Hiscott^11^. The reconstruction by Aksu and Hiscott (2022) suggests a Black Sea outflowing from 17.2 to 15.7 ka over the uppermost “Pleistocene delta Δ2” (− 55 m) as inferred from seismic reflection. (**c**) Black Sea freshwater outflowing (red, blue, and green) over the Bosphorus sill, as a result of (**a**) and (**b**). The black plain line marked with “B” represents an estimation (in mSv) by Chepalyga^13^. Black dots (marked with “a” and “b”) represent other two estimations for 15 ka at the Bosphorus Strait^31^. The horizontal plain lines represent the chronology reported in previous studies for Black Sea outflowing to the Marmara Sea (purple) and the Aegean Sea (orange). Blue patches represent the scenarios of the present study carrying out hosing experiments with a regional oceanic model: “4mSvFIS” (12,614 km^3^ of additional freshwater over 100 years) and “17mSvFIS” (53,611 km^3^ over 100 years).

The reconstructed volume of ice in the Black and Caspian Seas catchments and the associated meltwater flux show several phases from 21 to 10 ka (Fig. 2a). Between 20.5 and 15 ka, the ice mass loss of 23% to 26% of its 20.5 ka total is equivalent for all ice-sheet reconstructions. The associated meltwater flux increases between LGM and 16.5 ka. The mean flux over this period is 1.7, 2.2 and 2.6 mSv for ICE-6G, GLAC-1D, and Patton, respectively. At the BA onset, it abruptly increases following the ice mass loss. The maximum meltwater flux at that period is 17, 29 and 37 mSv for ICE-6G, GLAC-1D, and Patton, respectively. The flux then decreases until the end of the YD with a final increase until the FIS vanishes completely. The meltwater flux obtained here, especially for the BA, can be compared to those established in previous studies. The value of 50,000 m^3^ s^−1^ transferred from the Caspian Sea to the Black Sea (50 mSv) is based on lake levels and stratigraphy during the early and middle stages of deglaciation (17 to 14 ka)^13^ and is higher than the first pulse indicated by our reconstruction. Another study based on estimated river paleodischarge and ice sheet flow estimated a Caspian flooding to the Black Sea of ~ 23,000 km^3^ over 20–30 years (~ 29 mSv) around 15.5 ka^12^, which fits with the magnitude of our reconstructed meltwater fluxes.

The Black Sea water budget throughout the deglaciation is sensitive to P–E (positive P–E for TraCE-21 ka and negative P–E for MIROC4m, Supplementary Information). We evaluated in the Supplementary Information the likeliness of each combination. The most likely combination involves the average of P–E between TraCE-21 ka and MIROC4m, as shown in Fig. 2b. The BSL and associated volume changes show different behaviors depending on the ice sheet reconstructions. All the reconstructions have a rapid transgression phase starting from the LGM BSL (~ − 105 m^29,30^). Once the Bosphorus sill depth is reached for ICE-6G, the reconstruction, weighted by Black Sea P–E, shows overflowing from 19 to 10 ka. For GLAC-1D and Patton, the estimated BSL is punctuated by several regression and transgression phases. From 17 ka onwards, GLAC-1D experiences a first regression up to − 90 m at 16.5 ka, a second regression up to − 70 m at 15 ka and a last one at 12.5 ka up to 70 m. Patton shows a first regression phase up to − 120 m at 15 ka and a second up to − 90 m at ~ 12 ka.

To evaluate the likeliness of these scenarios in terms of sea-level amplitude, we represent the BSL as reconstructed by Lericolais et al.^29^, Genov^30^ and Aksu and Hiscott^11^. According to Lericolais et al., after a transgression phase (starting from − 105 m during the LGM), the BSL reaches the Bosphorus sill (close to − 37 m) from 16.5 to 13 ka, followed by a regression phase from 13 to 10 ka. According to Genov, after a long transgression phase, the BSL would reach the Bosphorus sill depth at 11.7 ka until 10.5 ka. According to Aksu and Hiscott^11^, the LGM BSL is higher (− 90 m). From 17.2 to 15.7 ka, Black Sea outflows over the uppermost “Pleistocene delta Δ2” (− 55 m)^11^ of the Bosphorus sill, and after 15.7 ka a regression phase occurs up to − 110 m around 14.7 ka. Then, the BSL increases until 11 ka when it reaches the shallowest sill depth. Our BSL based on ice sheet reconstructions shows a different timing than that of the previous reconstructions. However, the amplitude is comparable, especially for the regression phases.

Finally, we estimate the freshwater flux overflowing the Bosphorus sill (Fig. 2c). Between 20.5 and 15 ka, the average flux is 1.3, 1.9, 2.2 and 1.7 mSv for ICE-6G, GLAC-1D, Patton, ICE-AVERAGE, respectively. Abrupt melting occurs at ~ 14.5 ka for Patton et al., 14 ka for ICE-6G and 14.2 ka for GLAC-1D. The associated flux is 16, 28, 34 and 19 mSv for ICE-6G, GLAC-1D, Patton, ICE-AVERAGE, respectively. This event is followed by a quasi-continuous decrease reaching another peak (11.5 ka in Patton et al., and 11 ka for ICE-6G, GLAC-1D and ICE-AVERAGE) until the ice sheet vanishes completely. Over the period 11–10 ka, the average flux is 3.2, 2, 4.6 and 3.3 mSv for ICE-6G, GLAC-1D, Patton, ICE-AVERAGE, respectively.

Chepalyga (2007) estimated a flux of 60,000 m^3^ s^−1^ (60 mSv) from the Black Sea to the Marmara Sea, between 17 to 14 ka^13^ which is higher than our suggested flux. Another study using hydrological data and theoretical considerations of hydrometeorological watershed^31^ conditions shows a 15-ka pulse of either 200 km^3^ year^−1^ (~ 6.3 mSv) or 450 km^3^ year^−1^ (14.3 mSv) depending on the methodology. The second estimation agrees with the 14-ka peak in ICE-6G.

In terms of timing, at the early deglaciation stage, our reconstruction indicates an overflowing between 19.5 and 16.5 ka while most of the previous proxy-based studies suggest the 16.6–14.7 ka range. The BA meltwater pulse is inexistent in previous studies, mainly because of higher evaporation in the Black Sea and its catchment during this period. However, regarding the previous flux quantifications^13,32^, it is likely that our calculation of the BA-peak equates with the 16.6–14.7 ka outflowing phase. The post YD-peak, based on our calculations and previous studies would be the most likely outflowing phase.

We therefore propose an ensemble of forcing conditions to mimic different continental freshwater perturbations of the EMS. They constitute compromises between our climate forcings for the Early Holocene, our computation limitations, and our estimates of Black Sea outflow. We target the overflows during the BA with the “17mSvFIS” experiment and after the YD (“4mSvFIS”, Fig. 2c). In addition, we suggest the Nile River reached its highest activity after the YD due to last African Humid period^33,34^ and propose the “4mSvFIS + 9kNile” experiment. After the FIS melted completely, we also simulate “9kNile”, where the Nile River is considered to be the major freshwater contributor to the EMS.

### Impact on the EMS assessed with high-resolution modeling

**Figure 3:**
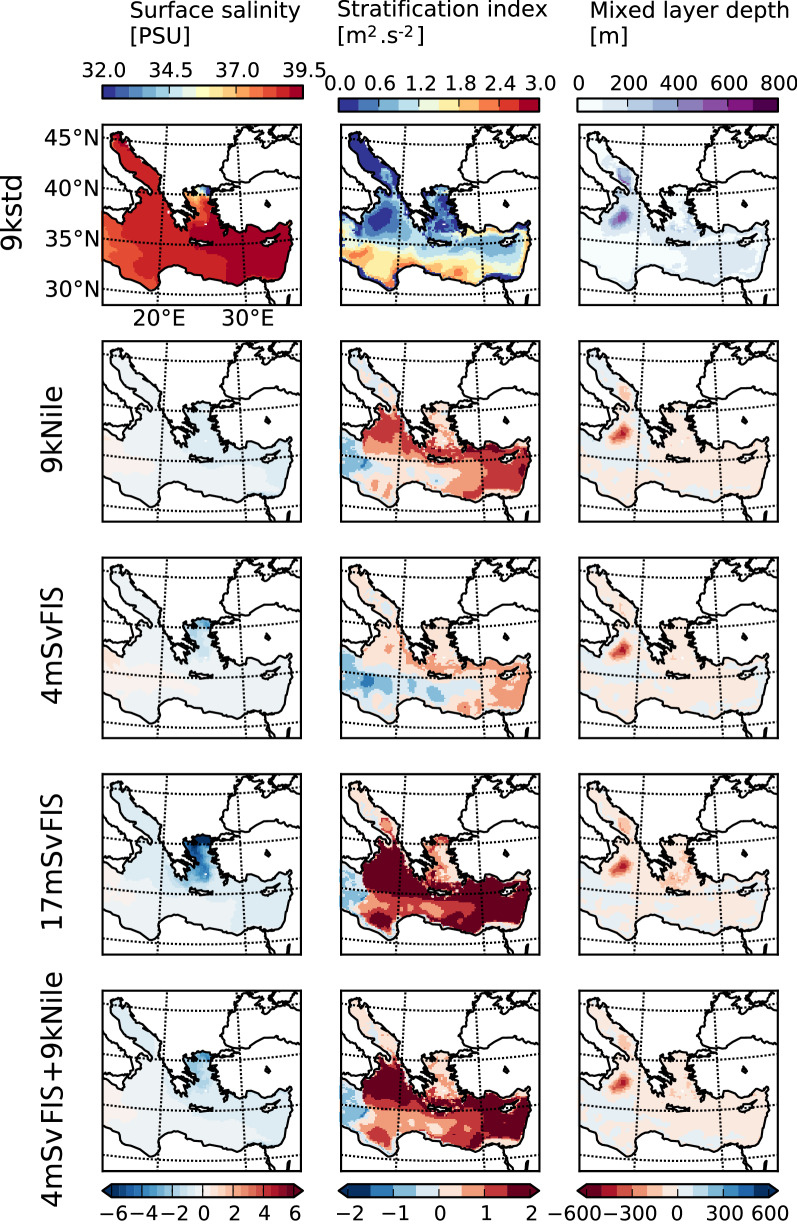
Output of the oceanic model: from left to right, columns are sea surface salinity (PSU), index of stratification (IS, m^2^ s^−2^), and mixed layer depth (m). The top row shows their climatological distributions in the “9kstd” simulation, and the remaining rows (from top to bottom) show the anomalies in each of the perturbation simulations. All simulations are at equilibrium. Figures show the average over the last 10 years. Figure have been generated using the Basemap package from Matplotlib (https://matplotlib.org/basemap).

The Early Holocene standard experiment (“9kstd”) simulates high SSS (38 to 39 PSU, increasing eastward) in the EMS due to high evaporation (Fig. 3). The stratification index (IS) and the mixed-layer depth (MLD) both depict areas of convection in the Aegean Sea (~ 0.3 m^2^ s^−2^ over the sea and 160–200 m in the south), North Levantine (0.6 m^2^ s^−2^ and 100–200 m), south Adriatic (0.6 m^2^ s^−2^ and between 400 and 500 m) and north Ionian Seas (0.3 m^2^ s^−2^ and up to 700 m). These surface and subsurface behaviors and properties are characteristic of the EMS in Mediterranean models without any massive freshwater input under either Early Holocene atmospheric conditions^4^ or present-day conditions^4,35–37^. They are quite close to data observed for the present day^38,39^.

The Black Sea outflow experiments (“4mSvFIS” and “17mSvFIS”) significantly reduce the SSS in the Northern Aegean Sea (− 2.1 and − 7 PSU for “4mSvFIS” and “17mSvFIS”, respectively, with a north–south gradient, Fig. 3 and Table 1). The freshening also impacts the Eastern Levantine (− 0.3 and − 0.5 PSU for “4mSvFIS” and “17mSvFIS”, respectively) and the Adriatic (− 0.2 PSU for “4mSvFIS” and “17mSvFIS”). “9kNile” shows a negative SSS anomaly that spreads westward following the coastline quite uniformly (− 1.7 PSU) over the most eastern part of the Levantine, and then over the Aegean (− 0.6 PSU in the Northern part), Northern Ionian, and Adriatic Sea (− 0.3 PSU). The combined experiment, “4mSvFIS + 9kNile”, depicting the additive effect of “4mSvFIS” and “9kNile”, produces a decrease in SSS at the Nile mouth (East Levantine: − 1.7 PSU) and over the Aegean Sea (northern part: − 2.6 PSU) and restricted to − 0.5 PSU elsewhere (with a similar pattern as “9kNile”).

**Table 1:** Values of SSS (in PSU) anomaly for each ORCM experiments (vs “9kstd”), and 11 vs 10 ka SSS anomaly at SL152^15^.

Sea surface salinity anomalies (PSU)	North aegean (40.3 N–40.9 N, 24.0 E–26.4 E)	East levantine (31.6 N–32.2 N, 30.8 E–32.0 E)	Adriatic (40.5 N–45.5 N, 12.0 E–19.0 E)	Sicily strait (36.4 N–37.6 N, 10.8 E–13.2 E)
4mSvFIS vs 9kstd	− 2.1	− 0.3	− 0.2	+ 0.5
17mSvFIS vs 9kstd	− 7.0	− 0.5	− 0.8	+ 0.2
9kNile vs 9kstd	− 0.6	− 1.7	− 0.3	+ 0.4
4mSvFIS + 9kNile vs 9kstd	− 2.6	− 1.7	− 0.5	+ 0.3
Herrle et al.: 11–10 ka at SL152	− 1.5			

Black Sea outflow experiments indicate a large decrease in oceanic convection potential and deep-water formation, characterized by increasing IS. In “4mSvFIS”, the Aegean, Eastern Levantine, South Adriatic, and North Ionian show a similar increase between + 1 and + 1.5 m^2^ s^−2^, whereas the western Levantine and Northern Ionian show decreasing IS (as much as − 1.5 m^2^ s^−2^, due to the slowdown of the zonal overturning circulation transporting low-salinity water masses from the western basin). In “17 mSv”, almost the entire EMS shows an increasing IS (up to + 2 m^2^ s^−2^ more pronounced over the Eastern Levantine, South Aegean, and Northern Ionian). Consequently, all intermediate and deep-water formation spots are affected in almost equal measure in “4mSvFIS” and “17mSvFIS”. The MLD is lifted up to − 100/− 200 m in the Aegean Sea, − 100 m in the Levantine (over a larger area in “17mSvFIS”) and − 500 m in the Southern Adriatic/Northern Ionian (also a more extended area in “17mSvFIS”). In “4mSvFIS + 9kNile”, the spatial structure and intensity of the IS anomaly is similar to “17mSvFIS”, although less extensive with an average value of ~ 1 m^2^ s^−2^. The MLD reduction pattern and values in this simulation are similar to those of “4mSvFIS” and “17mSvFIS” but are more widespread over the easternmost coast.

Placing these results in the context of the last deglaciation, the “9kstd” simulation represents the reference state. The anomalies of this simulation are compared to “17mSvFIS” where the flux linked to the BA meltwater pulse has a decreased ventilation mainly in the Adriatic/Ionian basins compared to the “4mSvFIS”. The remaining flux (~ 4 mSv) is superimposed on the Nile effect “4mSvFIS + 9kNile”) between the YD and the end of the deglaciation. In “4mSvFIS”, the SSS decrease is comparable to the decrease observed in SL152^15^ between 11 and 10 ka (simulation: − 2.1 PSU, observations: − 1.5 PSU, Table 1). After the FIS completely vanishes, the Nile in “9kNile” becomes one of the major sources of freshwater continuing to perturb the convection and ventilation of the EMS.

## Discussion

Black Sea outflow may affect the EMS at the BA onset and after the YD, although proxy-based data are not unanimous in confirming this north freshwater influence. How reliable are these experiments documenting extensive meltwater influx to the Aegean Sea and the EMS? The main argument against meltwater affecting the Aegean Sea (and the Marmara Sea) is the relatively weak δ^18^O depletion recorded in planktonic foraminifera which would belie meltwater intrusion.

**Figure 4:**
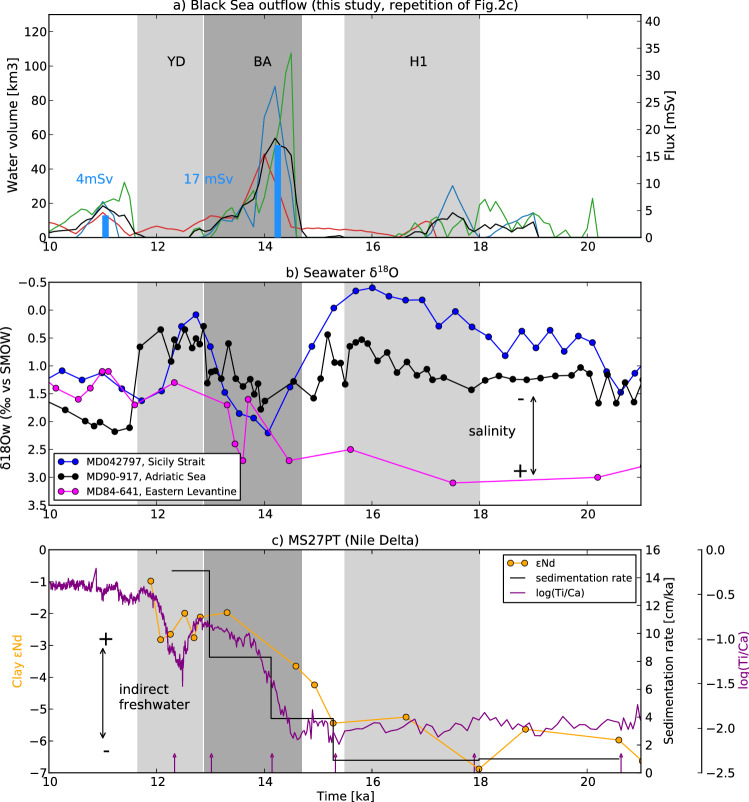
(**a**) Black Sea outflow chronology as calculated in this study (repetition of Fig. 2c). (**b**) δ^18^O_w_ from 21 to 10 ka in marine cores MD90-917 (41°17′N, 17°36′E; water depth: 1010 m, South Adriatic Sea^55^), MD07-2797 (36°57′N, 11°40′E; water depth: 771 m, Strait of Sicily^56,57^) and MD84-641 (33°02′N, 33°38′E; water depth: 1375 m, Eastern Levantine basin^58^). (**c**) Sedimentation rate, clay-εNd (fraction lower than 2 micron) in MS27PT (31°47′N, 29°27′E; water depth: 1389 m, Nile delta), decimal logarithm of the Ti/Ca ration scanned with XRF in MS27PT.

To discuss this issue, we compare our Black Sea outflow estimations with proxy data from the EMS. First, we calculate the sea-water δ^18^O (δ^18^O_w_), an estimate of the freshwater effect on three marine cores over the Central Mediterranean (MD042797, Sicily Strait), and the EMS (MD90-917, Adriatic Sea and MD84-641, Eastern Levantine) (Fig. 4b). Second, we document the chronology of Nile activity, the major freshwater contributor to the EMS, based on the deltaic core, MS27PT, from the middle stage of deglaciation to the Early Holocene. The reliability of the sediment core MS27PT in monitoring past Nile hydrology and reconstructing the African Humid Period in the Nile basin has been demonstrated in several sedimentology and geochemistry studies^34,40,41^. The ratio of Ti/Ca reflects the relative proportions of Nile terrestrial versus marine inputs at high temporal resolution. The sedimentation rate coupled with neodymium isotopes (expressed as εNd) are tracers of fluvial Nile activity (cf “Materials and methods”).

From 20 to 10 ka, δ^18^O_w_ records from the Sicily Strait and the South Adriatic Sea display a similar pattern indicating surface waters salinity fluctuations. An initial δ^18^O_w_ decrease between 20 and 16 ka marks the late glacial period followed by a deglaciation rise in δ^18^O_w_, more pronounced in the Sicily Strait, until the start of the BA at 14 ka. Between 14 and 12 ka, δ^18^O_w_ values decrease before rising until the onset of the Holocene at 11.7 ka. A final drop in δ^18^O_w_ characterizes the Early Holocene in both basins until 10 ka, suggesting the arrival of less saline surface waters. In contrast, in the Eastern Levantine, the δ^18^O_w_ record displays different behavior marked by a progressive decreasing trend (by 2.5 ‰) that lasted from 17 ka until 10 ka.

Between 14.8 and 5.5 ka, during the AHP, the northward migration of the rainfall belt in Africa enhanced precipitation in North Africa^34,42–44^. The massive reactivation of fluvial systems led to intensified precipitation in the Nile catchment and a massive inflow of freshwater to the EMS. In a recent study^41^, the record of suspended matter discharge from the Nile floods, indirectly linked to the freshwater input of the Nile, has been revisited using the Nd isotopes measured on the clay fraction to improve the reliability of the Nile fluvial activity records (cf “Materials and methods”).

The downcore evolution of clay-εNd is in phase with the ratio of Ti/Ca as well as with sedimentation rates (Fig. 4). This reflects low Nile fluvial activity during the LGM and the HS1. From 14.8 ka onwards, the three proxies agree and indicate a progressive increase in Nile fluvial activity, interrupted by the YD. This onset of Nile activity at 14.8 ka is also concomitant with an increase in organic component indicating higher terrigenous input from the Nile basin^34^.

Combining these proxies, we can conclude that Nile River intensification resulting from the AHP enhancement started between 15.7 and 14.5 ka and reached its highest activity between 12 ~ 10 and 6 ka. The δ^18^O_w_ decrease observed throughout deglaciation in the Eastern Levantine appears to face with our calculations of Black Sea freshwater outflow: δ^18^O_w_ decrease starts from 17 ka at the peak of the first phase which occurred between 19 and 16.5 ka. The decrease then became more pronounced from 14.5 ka with the BA-peak.

In contrast, in all freshwater scenarios, our results indicate a salinity increase in the Western Ionian and the Strait of Sicily (Fig. 3 and Table 1). Moreover, δ^18^O_w_ fluctuates more in the Adriatic and Sicily cores than in the Eastern Levantine core. The salinity increase in the ORCM in the Sicily is due to the reorganization of surface currents following massive freshwater inputs^45^. Thus, another pattern can be expected, consistent with the increase of δ^18^O_w_. The relative large δ^18^O_w_ increase in the Sicily Strait between ~ 16 to 14 ka could coincide with BA-peak meltwater. In the Adriatic, although the ORCM indicates homogenous salinity decrease, the IS differs locally (increase to decrease stratification compared to “9kstd” Fig. 3), especially at the location of the MD84-641core. Here, δ^18^O_w_ increase between 15.5 and 14 ka might coincide with the BA and the end of the YD when δ^18^O_w_ abruptly increased conjointly with the Black Sea outflow. However, a local freshwater explanation (the melting of the Alps, for instance) is more likely.

In the North Aegean Sea, the reconstructed SSS in the SL152 core shows a decrease between 11 and 10 ka similar to the salinity anomaly in the “4mSvFIS” experiment and is assumed to reflect the Black Sea outflowing during this period (SL152: − 1.5 PSU, “4mSvFIS”: − 2.1 PSU).

On the other hand, some studies propose that the freshening of the EMS occurring during the HS1 is due to the increasing exchange rate at the Strait of Gibraltar since the LGM^4,46^. Moreover, the rapid sea-level rise, especially during meltwater pulses, invigorated the water mass exchange at the Strait of Gibraltar, thus enhancing the freshening of the EMS^46^. The global sea-level rise is also consistent with the δ^18^O_w_ decrease. Our study does not refute this hypothesis but complements this approach with additional evidence on regional freshwater input.

Finally, we focus on ventilation which is sensitive to freshwater perturbation through water stratification changes. δ^13^C records in benthic foraminifera can give some insights in this regard, being a proxy of ventilation and of biological productivity. Results of benthic foraminiferal δ^13^C from various sites in the EMS reveal a decreasing trend in ventilation from the end of H1 until 9 ~ 8 ka, with a rapid drop during the BA and after the YD^4,18,20,47^. In our simulations, the impact on the MLD, and, thus, on the intermediate and deep ventilation, is quite similar, i.e., the enhanced freshwater input from the Black Sea has the same “dynamic” impact as the enhanced Nile input. The δ^13^C drop during the BA would be in line with our reconstructed Black Sea outflow. Nevertheless, the post-YD δ^13^C drop is more difficult to link to the Black Sea outflow than to the intensified Nile fluvial activity that occurred at the same period^41^. Moreover, our experimental configuration does not permit us to indicate if the decrease in ventilation following Black Sea outflow could have persisted for thousands of years, thus allowing water stagnation and low benthic foraminiferal δ^13^C.

## Conclusions

We identify in this study a plausible role of FIS melting in the freshening of the EMS through the Black Sea. The FIS evolution shows nonlinearity and the meltwater flux is punctuated by at least three phases during the deglaciation.

We quantify the potential effect of FIS meltwater on the EMS. Our work reveals a high sensitivity in the EMS circulation and shows that Black Sea freshwater input played a significant role in reducing the SSS and ventilation of the EMS. This effect is important when combined with enhanced freshwater input into the EMS due to a strengthened African Monsoon, leading to an additional decrease in salinity and convection in the EMS.

Our results are consistent with proxy data recorded in the Black Sea and the Marmara Sea for the early and late deglaciation but not during the BA period. Moreover, a certain divergence remains with marine proxies in the Aegean Sea and more generally in the EMS: the enhanced freshwater during the deglaciation may not be attributed to meltwater from the Black Sea but rather to the global sea-level rise. Nevertheless, our work complements the existing hypothesis which relies on long-term dynamics of the Atlantic Ocean and on variations in the water properties at the Strait of Gibraltar.

The Black Sea hydrology throughout the last deglaciation deserves more careful analysis of FIS melting, the changes in precipitation-driven river discharges and the complex geomorphology of the Bosphorus and Dardanelles straits. A joint inventory of these contributions considering both a modeling approach and proxy evidence is crucial to accurately quantify the Black Sea outflow to the Marmara and the Aegean Sea. Usually neglected, they may additionally contribute to the S1 preconditioning in the EMS, as is suggested for the Aegean Sea^11^.

## Materials and methods

### Estimation of the meltwater influx to the Black Sea and Caspian Sea catchments

Three sources are used to estimate the meltwater flux. The first is the ICE-6G reconstruction^26^ recording the global ice sheet evolution from 24 to 8 ka with a 500-year time step between 21 and 10 ka. ICE-6G uses Global Positioning System observations augmented by additional space geodetic constraints such as sea-level from complementary systems. The second source is the GLAC-1D reconstruction^27^ which is based on a set of glaciological models derived from plausible climate forcing of PMIP1 and PMIP2 LGM simulations. GLAC-1D data are provided with a frequency of 100 years. For the sake of simplicity, we extract the ice volume with a time step of 500 years from 21 to 10 ka. The time step is refined to 200 years between 16 and 13 ka, with the first melting maximum expected during the BA. For both ICE-6G and GLAG-1D, we adjust the thickness of the FIS before computing the ice volume taking the isostasy into account. We then estimate the potential flux of meltwater directed to the Caspian and Black Seas through the Volga, Don, and Dnieper rivers. The third source is the reconstruction by Patton et al.^25^, recording the FIS evolution with a 50-year time step based on a 3-dimensional thermomechanical ice sheet model. In Patton et al.’s model, the temperature and precipitation adjust to the evolving ice sheet surface through the applied lapse rates derived from multiple regression analyses of meteorological observations. Moreover, the regional reference climates and associated forcing are tuned independently for each of the three major ice accumulation centers (of the Eurasian ice sheet complex) to account for the large variation in climate regimes across the Eurasian domain. Patton et al.’s reconstruction shows the entire FIS chronology (1 dimension), with a maximum volume of 3.7 10^6^ km^3^ reached at 22.8 ka. Data from ICE-6G and GLAC-1D (providing 3-D reconstruction) are processed with Integrated Geo Systems to show the expected ice volume and meltwater directed to the Black Sea and Caspian Sea catchments. From 23 ka, both GLAC-1D and ICE-6G show a maximum volume of 1.2 10^6^ km^3^ (at 21 ka for GLAG-1D and 20 ka for ICE-6G) in the selected area. To ensure a direct comparison between the three datasets, we simply divide Patton et al.’s data by a factor of 3 in our calculation.

### Estimation of the Black Sea outflow

From the FIS reconstructions, we evaluate the BSL evolution from the LGM and the likely freshwater outflow over the Bosphorus sill to the Marmara Sea. First, we use recent simulations of the last deglaciation from MIROC4m^48^, an atmosphere–ocean coupled general circulation model (AOGCM), and TraCE -21ka^49^ to give an estimation of precipitation (P) and evaporation (E) over the Black Sea during this period. The simulation of the last deglaciation was performed under fixed LGM ice sheets and time-evolving orbital parameters. The TraCE-21 ka dataset contains output from the full TraCE-21 ka simulation from 22,000 years before present (22 ka) to 1990 CE. The model (CCSM3) was forced with transient greenhouse gas concentrations and orbitally driven insolation changes. Transient boundary conditions include the ICE-5G ice sheets extent and topography and changing paleogeography with sea level rises from its Last Glacial Maximum level to modern level. The P–E from MIROC4m and TraCE-21 ka is presented in the Supplementary Information. Second, we represent the BSL reaching the Bosphorus sill and the associated volume changes. As the Bosphorus sill depth during the last deglaciation is open to debate^11^, for the sake of simplicity and to avoid overestimation, we consider that the Black Sea outflows when the sea level reaches the shallow sill in the Southern Strait of Bosphorus (− 37 m today). To estimate the volume needed for outflow to occur from the LGM BSL, we take account of the modern Black Sea area (436,400 km^2^). As estimating BSL is highly sensitive to P–E, we consider every combination, including a bias correction of P-E relative to modern observations. To evaluate the plausibility of each scenario, we compare them with BSL reconstructions^11,29,30^. As two reconstructions^29,30^ are in ^14^C age, we use the Black Sea reservoir age estimated by Soulet et al.^5^ to estimate the calibrated age (shown in the Supplementary Information). Finally, we choose the P–E combination that reflects a sea-level amplitude similar to the reconstruction and deduce the associated outflow. The selected scenario takes the average of MIROC4m P–E (bias corrected, P–E negative) and TraCE-21 ka (unbiased, P–E positive).

### Description of the oceanic model

The Ocean Regional Climate Model, NEMOMED8^50,51^, is the Mediterranean version of the NEMO ocean modeling platform^52^. The horizontal domain includes the whole Mediterranean Sea and the near Atlantic Ocean serves as a buffer zone. The horizontal resolution is 1/8° in longitude and 1/8°cosφ in latitude, approximately 9 km to 12 km from north to south. Vertically, the model has 43 layers of inhomogeneous thickness. Represented in the model are 33 river mouths. The Black Sea is considered to be a river flowing into the Aegean Sea. All simulations are analyzed at their equilibrium state over the last 10 years of simulation.

### Atmospheric forcing

The atmospheric forcing driving NEMOMED8 was derived from the Mediterranean regional simulation of the Early Holocene period (9.5 ka), corresponding to the last insolation maximum, performed with a sequential global-regional modeling procedure, LMDZ-NEMO-med-v1^28^. This architecture is based on the IPSL-CM5 global simulation (Coupled Model Intercomparison Project protocol)^53^. The 30-year atmospheric forcing is the same for each simulation and is used in a loop to force, with sea-surface temperature (SST), wind stress, P minus E, solar flux, and total heat flux, the 100-year Mediterranean oceanic circulation experiments.

### Freshwater forcing

The different simulations only differ in the fluvial input applied. “9kStd” is a virtual configuration with preindustrial river runoff^54^. “9kNile” uses the Early Holocene Nile enhancement provided by LMDZ-NEMO-med-v1^28^. This reconstruction uses the continental hydrological budget over the Nile catchment to provide a climatological signal. With this setting, the Early Holocene Nile has a mean runoff of approximately 15 mSv (3 mSv for the pre-damming value). For the Black Sea outflow experiments (“4mSvFIS”, “17mSvFIS”), we use fluxes as presented in the results. Freshwater perturbations have been applied at the Bosphorus Strait uniformly over the year.

### Sea surface water δ^18^O calculation

Three deep-sea cores have been selected for the δ^18^O (δ^18^O_w_) calculation: MD90-917 (41°17′N, 17°36′E; water depth: 1010 m^55^), MD07-2797 (36°57′N, 11°40′E; water depth: 771 m^56,57^) and MD84-641 (33°02′N, 33°38′E; water depth: 1375 m^58^) collected in the South Adriatic Sea, in the central part of the Strait of Sicily and in the Levantine basin, respectively.

SSS as expressed by local sea-water δ^18^O_w_ was determined following the method of Duplessy et al.^59^. Combining both planktonic foraminiferal δ^18^O and SST reconstructions^55^, we derived the sea-surface δ^18^O_w_ by solving the paleotemperature equation of Shackleton^60^:

$$T \, \left( {^{ \circ } {\text{C}}} \right) \, = \, 16.9 \, {-} \, 4.38\delta^{18} O_{calcite} - \delta^{18} O_{w} + \, 0.27) \, + \, 0.1\delta^{18} O_{foraminifera} - \delta^{18} O_{w } + \, 0.27)^{2} .$$

Variations in δ^18^O_w_ reflect both the global mean oceanic isotopic composition related to continental ice volume changes and local changes due to freshwater inflow variations and evaporation balance. Local δ^18^O_w_ changes were obtained by subtracting the effect of continental ice melting from global sea-water δ^18^O. The latter is assumed to be equal to the deglaciation sea level curve^61,62^ multiplied by a constant coefficient of ~ 1.05‰ as per Duplessy et al.^63^. We did not convert the δ^18^O_w_ values into salinity units because of uncertainty resulting from possible temporal changes in the slope of the δ^18^O_w_ /salinity relationship at the studied core site^64^. The accuracy of the δ^18^O_w_ estimates depends primarily on the SST estimates, given that there is a 0.07‰ error due to mass spectrometer measurements and the mean standard deviation on SSTs (~ 1 °C error for SST estimates would result in a 0.2 ‰ error in the calculated δ^18^O_w_ value).

### εNd, sedimentation rate and Ti/Ca evolution in MS27PT

The MS27PT sediment core (31°47′N, 29°27′E; water depth: 1389 m) located in the Nile deep-sea fan is directly influenced by Nile freshwater input and provides a continuous record of fluvial sediment discharge from the Nile River basin for the last 110 ka^40,41^. This is well illustrated by high-resolution XRF core scanner log (Ti/Ca) ratios which reflect varying proportions of terrestrial to marine inputs following the insolation. The Nile watershed is essentially composed of two main catchments, both of silicate lithology: the Ethiopian highlands, or traps, mainly composed of basaltic volcanic rocks and drained by the Blue Nile and the Atbara rivers and the Equatorial craton, mainly composed of gneiss and granulite rock and drained by the White Nile. Sediment Nd isotopic compositions (expressed using the epsilon notation εNd) are little affected by weathering processes and hence faithfully reflect geographical provenance and crustal age of the source rocks^65^. Thus, the Nile basin is well suited to isotopic tracer analysis because of the contrasting εNd signatures characterized by the Cenozoic Ethiopia traps (εNd ~ 0) and the Precambrian Central Africa Craton (εNd ~ − 30). The sediment loads of each tributary have a distinct Nd isotopic composition reflecting the geology of the catchments. Thus, temporal changes in both Nd signature and sedimentation rate allow changes in the terrigenous contribution relative to spatial precipitation changes^34^ to be reconstructed. Recently, the use of Nd isotopes focuses on the clay size fraction of the sediment (< 2 µm) in order to minimize potential complexities related to granulometric processes and mineral sorting occurring during sediment transport and deposition, as described in Bastian et al.^66^. Thus, the variation in the clay-εNd signal coupled with sedimentation rate can be interpreted as Nile fluvial activity change. At the scale of the Holocene and the last deglaciation, Bastian et al.^41^ found that the highest εNd values recorded during the AHP, from ~ 14 to ~ 8 ka BP, indicate high Nile fluvial activity with larger proportions of particles derived from the Ethiopian Traps. Indeed, the radiogenic clay-εNd (~ -2) are closer to values observed in the Ethiopian Traps (εNd 0 to 7). In contrast, during LGM, sediments are characterized by lower εNd values (-8) and low sedimentation rate, indicating reduced Nile fluvial activity.

## Supplementary Information

Supplementary Information.

## Data Availability

The ice sheet data set, the ORCM output and the marine proxy data used in this study are made available in a Zenodo repository (10.5281/zenodo.6496596). Additional data related to this paper may be requested from the authors.
