# Author Correction: Freshwater influx to the Eastern Mediterranean Sea from the melting of the Fennoscandian ice sheet during the last deglaciation

**DOI:** 10.1038/s41598-023-28159-1

**Published:** 2023-01-18

**Authors:** Tristan Vadsaria, Sébastien Zaragosi, Gilles Ramstein, Jean-Claude Dutay, Laurent Li, Giuseppe Siani, Marie Revel, Takashi Obase, Ayako Abe-Ouchi

**Affiliations:** 1 Laboratoire des Sciences du Climat et de l’Environnement, CEA-CNRS-Université Paris Saclay, Gif-sur-Yvette, France; 2 Atmosphere and Ocean Research Institute, The University of Tokyo, 5-1-5, Kashiwanoha, Kashiwa, Chiba 277-8568 Japan; 3 UMR-CNRS 5805 EPOC, Université de Bordeaux, Allée Geoffroy Saint-Hilaire, Pessac, France; 4 Laboratoire de Météorologie Dynamique, CNRS-ENS-Ecole Polytechnique-Sorbonne Université, Paris, France; 5 GEOPS, UMR 8148 Université Paris-Saclay, Orsay, France; 6 Université de la Côte d’Azur, CNRS, OCA, IRD, Geoazur, Valbonne, France

Correction to: *Scientific Reports* https://doi.org/10.1038/s41598-022-12055-1, published online 19 May 2022

The original version of this Article contained errors.

The authors omitted the below References, which is listed below as Reference 68 and 69.

68. Grosswald, M. G. Late Weichselian ice sheet of Northern Eurasia. *Quat. Res.*, **13**(1), 1–32 10.1016/0033-5894(80)90080-0 (1980).

69. Komatsu, G., Baker, V. R., Arzhannikov, S. G., Gallagher, R., Arzhannikova, A. v, Murana, A., & Oguchi, T. Catastrophic flooding, palaeolakes, and late Quaternary drainage reorganization in northern Eurasia. *Int. Geol. Rev.*, **58**(14), 1693–1722 10.1080/00206814.2015.1048314 (2016).

As a result, in the legend of Figure 1,

“Map of LGM ice caps including the Fennoscandia Ice Sheet^67^ and its southward drainage basin with major rivers indicated, including the Volga, Don, and Dnieper.”

now reads:

“Map of LGM ice caps including the Fennoscandia Ice Sheet^67^ and its southward drainage basin with major rivers indicated, including the Volga, Don, and Dnieper^68,69^.”

In addition, the original version of this Article contained an error in the legend of Figure 2.

“(**b**) Black Sea level (right axis, m, relative to modern sea level) and associated volume changes (left axis, km^3^) deduced from the Black Sea’s water budget with FIS meltwater input as shown in panel **a**), and the regional water flux P–E (precipitation-minus-evaporation) from two transient simulations of MIROC4m and Trace-21 ka. The water volume is expressed as the complementary volume needed to reach the Bosphorus sill at − 37 m, the level necessary for the Black Sea to outflow to the Mediterranean. The lowest BSL at the LGM is estimated to be – 105 m^29,30^ and the total volume for outflowing to occur is 29,675 km^3^ (light blue horizontal patch). Superimposed are BSL reconstructions by Lericolais et al.^29^, Genov^30^ and Aksu and Hiscott^11^. The reconstruction by Aksu and Hiscott (2022) suggests a Black Sea outflowing from 17.2 to 15.7 ka over the uppermost “Pleistocene delta Δ2” (− 55 m) as inferred from seismic reflection. The horizontal grey patch represents the broad range of the volume (29,675 km^3^) needed to outflow over the shallowest modern sill depth (− 37 m) from the lowest estimation of the BSL at the LGM (− 105 m).”

now reads:

“(**b**) Black Sea level (right axis, m, relative to modern sea level) and associated volume changes (left axis, km^3^) deduced from the Black Sea’s water budget with FIS meltwater input as shown in panel **a**), and the regional water flux P–E (precipitation-minus-evaporation) from two transient simulations of MIROC4m and Trace-21 ka. The water volume is expressed as the complementary volume needed to reach the Bosphorus sill at − 37 m, the level necessary for the Black Sea to outflow to the Mediterranean. The lowest BSL at the LGM is estimated to be − 105 m^29,30^ and the total volume for outflowing to occur is 29,675 km^3^ (light blue horizontal patch). Superimposed are BSL reconstructions by Lericolais et al.^29^, Genov^30^ and Aksu and Hiscott^11^. The reconstruction by Aksu and Hiscott (2022) suggests a Black Sea outflowing from 17.2 to 15.7 ka over the uppermost “Pleistocene delta Δ2” (− 55 m) as inferred from seismic reflection.”

The original Article has been corrected.

