# Supplementary material for: Freshwater influx to the Eastern Mediterranean Sea from the melting of the Fennoscandian ice sheet during the last deglaciation
Source: Sci Rep. 2022 May 19;12:8466. doi: 10.1038/s41598-022-12055-1 (PMC9120512; doi:10.1038/s41598-022-12055-1)
Supplement: Supplementary file 1 — Supplementary Information. [file 41598_2022_12055_MOESM1_ESM.docx]

**Supplementary Information for**

Freshwater influx to the Eastern Mediterranean Sea from the melting of the Fennoscandian ice sheet during the last deglaciation

Tristan Vadsaria*^1,2^, Sébastien Zaragosi^3^, Gilles Ramstein^1^, Jean-Claude Dutay^1^, Laurent Li^4^, Giuseppe Siani^5^, Marie Revel^6^, Takashi Obase^2^, Ayako Abe-Ouchi^2^

^1^Laboratoire des Sciences du Climat et de l'Environnement, CEA-CNRS-Université Paris Saclay, Gif-sur-Yvette, France

^2^Atmosphere and Ocean Research Institute, The University of Tokyo, 5-1-5, Kashiwanoha, Kashiwa, Chiba 277-8568, Japan

^3^UMR-CNRS 5805 EPOC, Université de Bordeaux, allée Geoffroy Saint-Hilaire, Pessac, France

^4^Laboratoire de Météorologie Dynamique, CNRS-ENS-Ecole Polytechnique-Sorbonne Université, Paris, France

^5^GEOPS, UMR 8148, Université Paris-Saclay, Orsay, France

^6^Université de la Cote d'Azur, CNRS, OCA, IRD, Geoazur, Valbonne, France

*Corresponding author: Tristan Vadsaria

**Email:**  [vadsaria@aori.u-tokyo.ac.jp](mailto:vadsaria@aori.u-tokyo.ac.jp)

**This file includes:**

Supplementary text

Figures S1 to S4

SI References

Supplementary Text

**Selection of the Black Sea outflowing scenario**

We firstly use the evaporation (E) and precipitation (P) as simulated by TraCE-21ka and MIROC4m to assess the atmospheric component of the water budget over the Black Sea. In Figure S1 are represented P, E and P-E of MIROC4m, TraCE-21ka, together with modern values as compiled by Romanou et al (2010)^1^ using different re-analyses. From 21 to 10 ka, MIROC4m has higher E (mean: 2.4 mm/day) than P (mean: 1.4 mm/day). P-E is negative (mean: -1.1 mm/day), decreases from 21 to 14ka and is quasi constant up to 10ka (10ka vs 21ka: -0.30 mm/day). On the contrary, TraCE-21ka shows that P (1.2 mm/day) is higher than E (0.6 mm/day) leading to positive P-E (0.6 mm/day), values quasi-constant over the deglaciation. For modern values, E (2.6 mm/day) is higher than P (1.6 mm/day), and P-E negative (-0.8 mm/day). To evaluate the impact of bias correction of MIROC4m and TraCE-21ka onto the Black Sea inflow and outflow, we also bias correct P-E regarding their modern simulated values (bias-corrected P-E, MIROC4m: -0.8 mm/day, TraCE-21ka: -0.5 mm/day).

We can now evaluate again the water budget of the Black Sea, but with glacial meltwater taken as runoff R, i.e., the meltwater flux derived from ice sheet reconstructions into the Black Sea, as estimated in Figure 1a of the main text. We considered a range of possible scenarios: P-E from modern observation, unbiased MIROC4m (Figure S2a) and TraCE-21ka (Figure S2b), and bias-corrected MIROC4m (Figure S2c) and TraCE-21ka (Figure S2d).

Finally, we evaluate the Black Sea level from the inflow, following the protocol described in the main text (Figure S3). We retained the scenarios that do not show Black Sea level regression lower than -120m (Figure S3c and h). Among the later scenarios, we choose the combination of P-E that reflect a similar sea-level amplitude than the reconstruction and deduce the associated outflowing flux (i.e., the remaining volume over the shallow modern sill in the southern Strait of Bosphorus). The selected scenario involves the average of MIROC4m P-E (bias corrected, P-E negative) and TraCE-21ka (unbiased, P-E positive) (Figure S3h). We do not choose the scenario associated with Figure S3c as it uses positive P-E (from TraCE-21ka): positive P-E is unlikely, at least in the middle stage of the deglaciation^2–4^, and it also leads to quasi-immediate and constant Black Sea outflowing through the last deglaciation.

Supplementary Figures


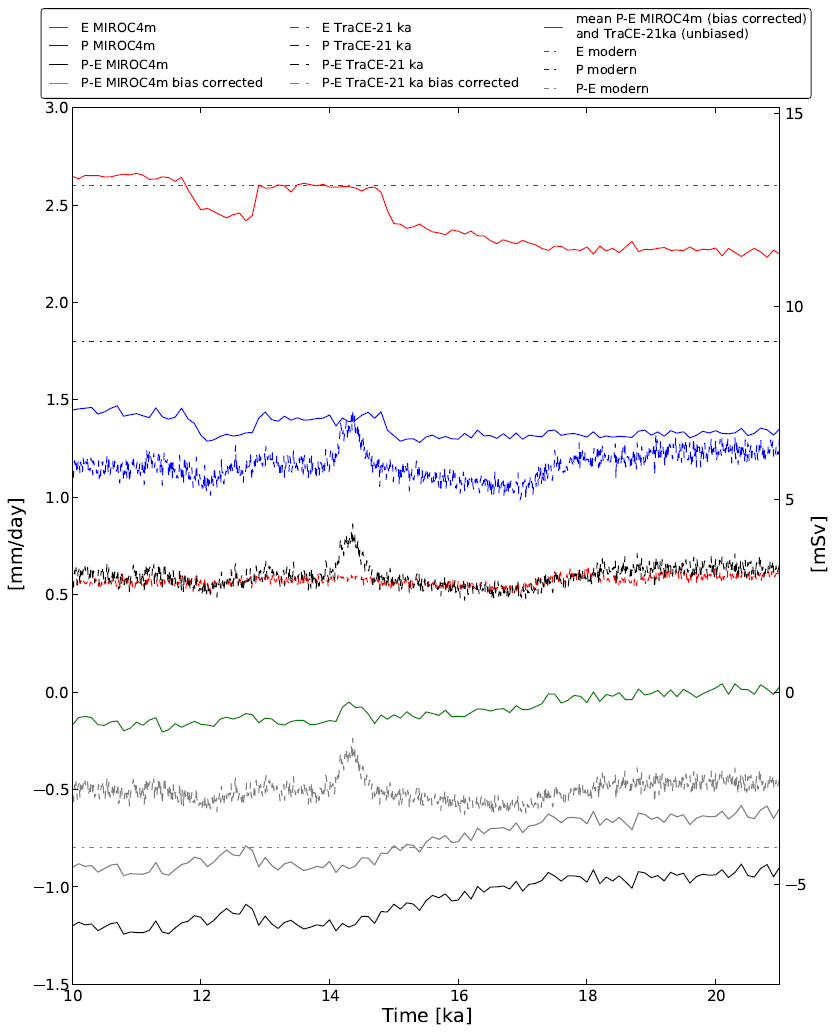


**Figure. S1.** Precipitation (P), evaporation (E) and P minus E from modern observations, MIROC4m and TraCE-21ka. For the transient simulations, unbiased and bias-corrected values are shown, as well as the average between MIROC4m (bias-corrected) and TraCE-21ka (unbiased), the finale combination retained for our study. Values are expressed in mm/day and in mSv (10^3^km^3^) using the modern Black Sea area (436 400 km^2^).


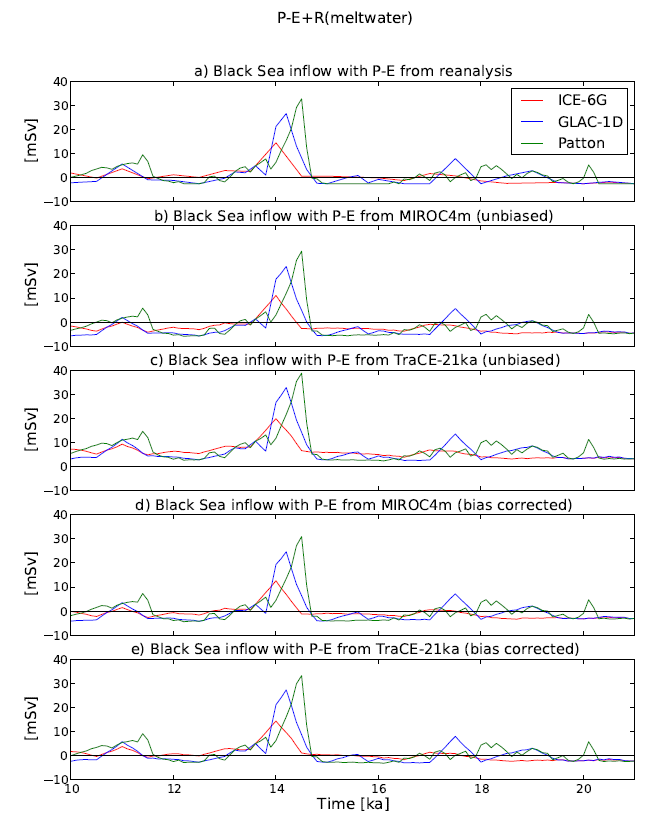


**Figure. S2.** P minus E plus meltwater runoff (R) in mSv, derived from ice sheet reconstructions (cf main manuscript and material and method section). Several P minus E scenarios are used as described in the supplementary text.


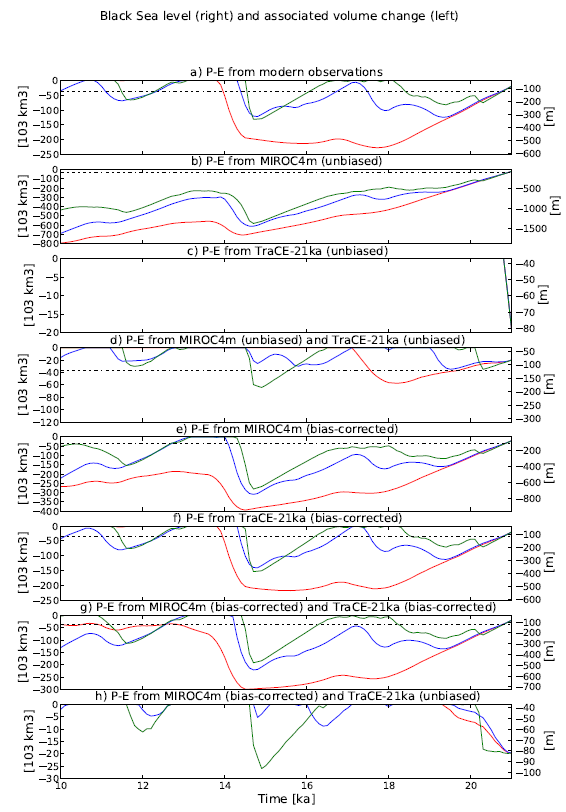


**Figure. S3.** Right axis, Black Sea levels deduced from Figure S2 for several P minus E scenarios. Calculated sea level starts from the lowest estimation of reconstructed LGM Black Sea level^5,6^. Horizontal dashed line represents the value y=120m. Left axis, equivalent water volume using modern Black Sea area (436 400 km^2^).


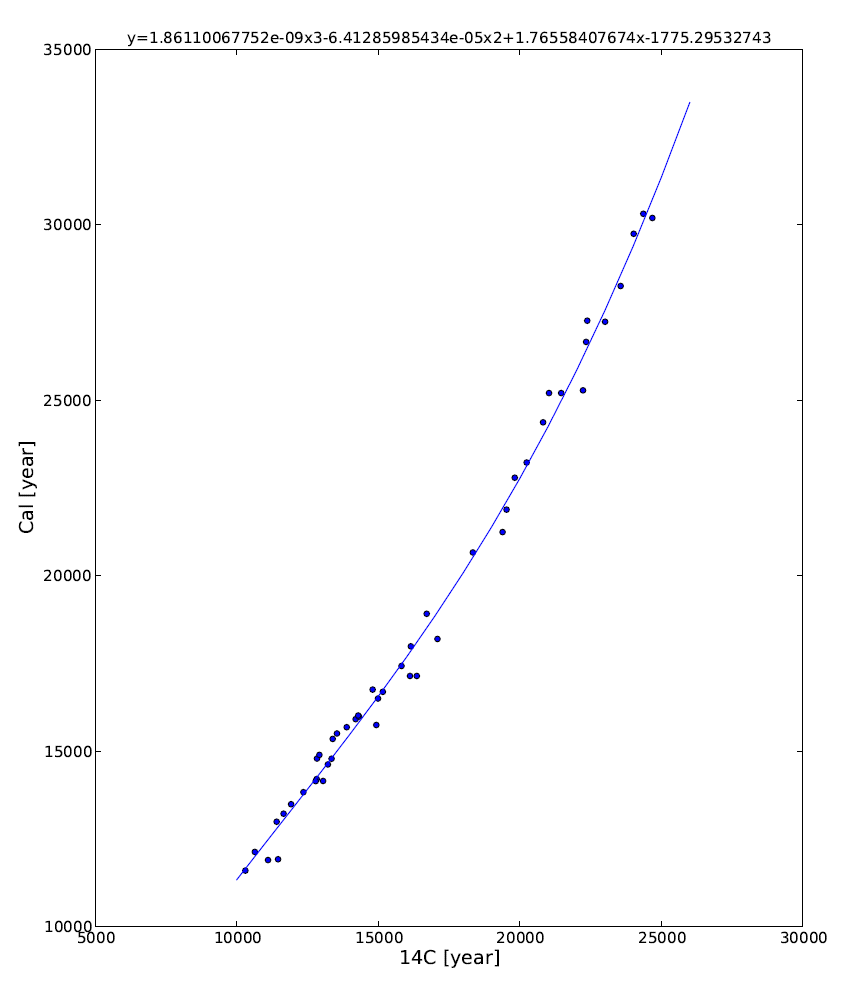


**Figure. S4.** Black Sea reservoir age from Soulet et al. (2011)^3^. Above is shown the regression fit.

**SI References**

1. Romanou, A. *et al.* Evaporation–Precipitation Variability over the Mediterranean and the Black Seas from Satellite and Reanalysis Estimates. *J. Clim.* **23**, 5268–5287 (2010).

2. Huang, Y., Zheng, Y., Heng, P., Giosan, L. & Coolen, M. J. L. Black Sea paleosalinity evolution since the last deglaciation reconstructed from alkenone-inferred Isochrysidales diversity. *Earth Planet. Sci. Lett.* **564**, 116881 (2021).

3. Soulet, G. *et al.* Black Sea “Lake” reservoir age evolution since the Last Glacial — Hydrologic and climatic implications. *Earth Planet. Sci. Lett.* **308**, 245–258 (2011).

4. Bahr, A. *et al.* Abrupt changes of temperature and water chemistry in the late Pleistocene and early Holocene Black Sea. *Geochemistry, Geophys. Geosystems* **9**, (2008).

5. Lericolais, G., Bulois, C., Gillet, H. & Guichard, F. High frequency sea level fluctuations recorded in the Black Sea since the LGM. *Glob. Planet. Change* **66**, 65–75 (2009).

6. Genov, I. The Black Sea level from the Last Glacial Maximum to the present time. *Geol. Balc.* **45**, 3–19 (2016).
